# Peritoneal mesothelioma causing small bowel obstruction in COVID-19 positive patient

**DOI:** 10.1093/jscr/rjac469

**Published:** 2022-10-23

**Authors:** Aakash Trivedi, Ahmad Hlayhel, Zamaan Hooda, Lindsey Foran, Scott Wessner

**Affiliations:** Department of Surgery, St. Joseph’s University Medical Center, Paterson, NJ, USA; Department of Surgery, St. Joseph’s University Medical Center, Paterson, NJ, USA; Department of Surgery, St. Joseph’s University Medical Center, Paterson, NJ, USA; Department of Surgery, St. Joseph’s University Medical Center, Paterson, NJ, USA; Department of Surgery, St. Joseph’s University Medical Center, Paterson, NJ, USA

## Abstract

Mesothelioma is a disease process that can present in a variety of locations including the pleural, peritoneum and pericardium. Malignant peritoneal mesothelioma has been known to be a particularly aggressive type of tumor. We report a case of a patient who presented with a small bowel obstruction whose pathology revealed peritoneal malignant mesothelioma.

## INTRODUCTION

Mesothelioma is a rare and lethal neoplasm of serosal membranes, most commonly affecting the pleura, and less commonly the peritoneum, pericardium and tunica vaginalis. Malignant peritoneal mesothelioma is a more aggressive variant of this disease, accounting for ~10% of cases of mesothelioma. There is a strong association between asbestos exposure and mesothelioma; other risk factors include radiation and mineral fiber exposure. After initial exposure to asbestos, mesothelioma can take 20–50 years to develop symptoms, making it difficult to diagnose. Peritoneal mesothelioma is frequently misdiagnosed due to its presentation of symptoms, which may overlap with neoplasms such as adenocarcinoma of the ovary and other abdominal organs. In this case, we present an 81-year-old male with a small bowel obstruction whose final pathology showed peritoneal malignant mesothelioma of the epithelial subtype.

## CASE PRESENTATION

**Figure 1:**
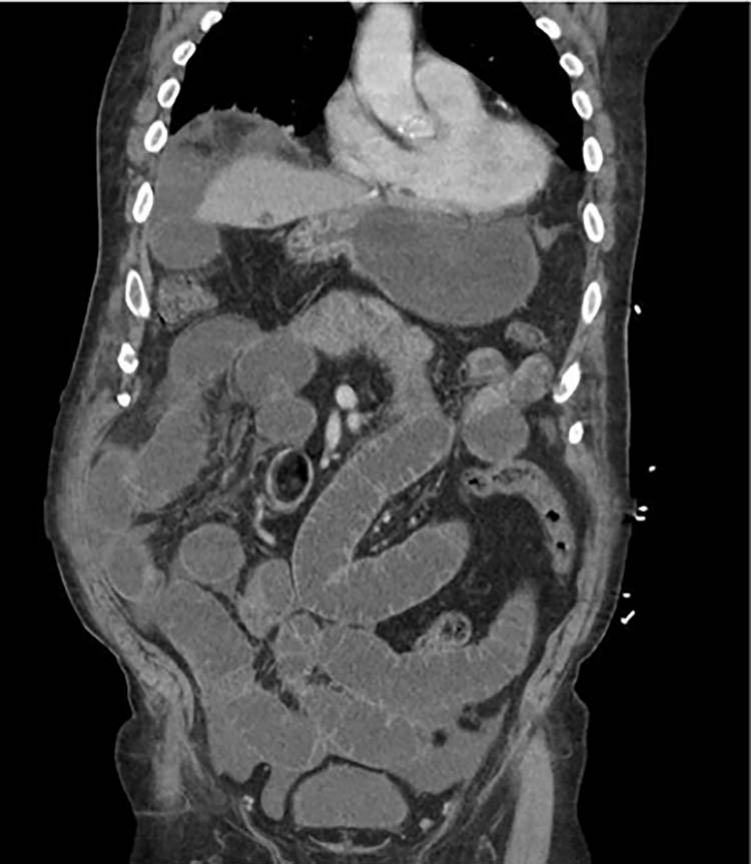
Abdominal CT scan on patient initial presentation.

**Figure 2:**
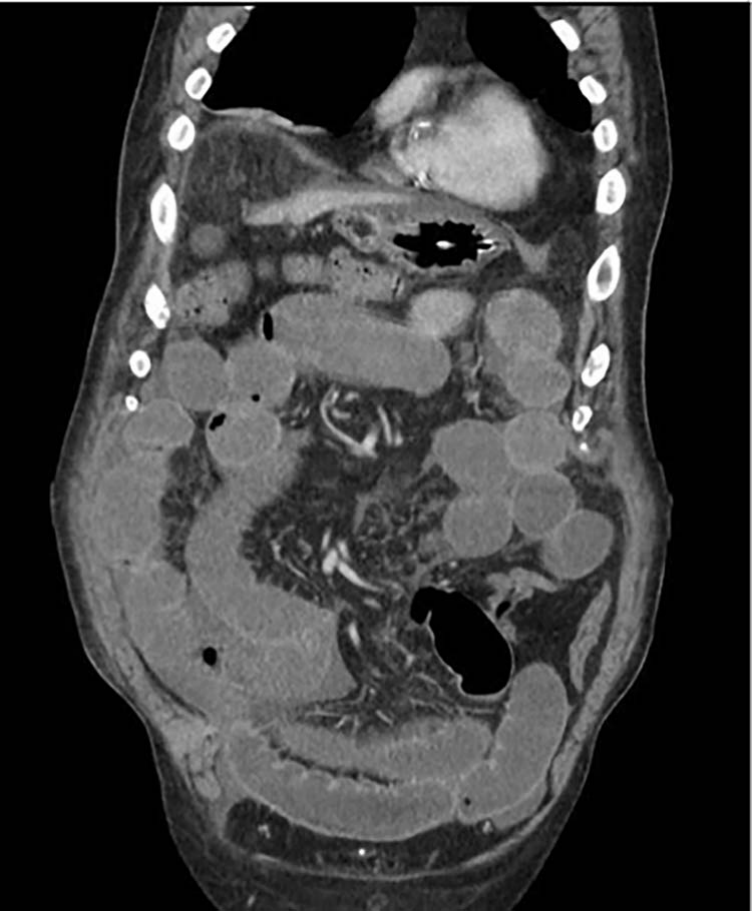
Repeat CT scan after attempted conservative management.

An 81-year-old male with past medical history of coronary artery disease, peripheral arterial disease, hypertension, dyslipidemia, hypothyroidism, benign prostatic hyperplasia, osteoarthritis, sleep apnea, gout and 10-pack year smoking history presented with complaints of abdominal distention and bloating. Patient had been suffering from recurrent bouts of constipation for ~2 weeks with last bowel movement noted to be over 2 days prior to presentation. Patient also complained of numerous episodes of nonbilious non-bloody vomiting. Abdominal computed tomography (CT) scan (Fig. 1) presented findings of a small bowel obstruction with transition point at the level of the terminal ileum. Notably, patient was also positive for coronavirus disease 2019 (COVID-19) infection. General surgery was consulted, and patient was initially treated with non-operative management with nasogastric tube and serial abdominal exams. However, a repeat abdominal CT scan (Fig. 2) after 6 days of attempted conservative management showed persistent diffuse small bowel distention and increased ascites. This combined with the fact that the patient had no prior colonoscopies or abdominal surgeries prompted surgical exploration. Patient underwent exploratory laparotomy; intraoperatively, he was found to have a palpable mass at the terminal ileum. A right hemicolectomy with a side-to-side functional end-to-end ileocolonic primary anastomosis was performed. Final pathology revealed diffuse peritoneal malignant mesothelioma with no nodal involvement and negative margins.

## DISCUSSION

Mesothelioma is a rare and lethal neoplasm of serosal membranes, most commonly affecting the pleura, and less commonly the peritoneum, pericardium and tunica vaginalis. Malignant peritoneal mesothelioma is a more aggressive variant of this disease, accounting for ~10% of cases of mesothelioma. Rates of peritoneal mesothelioma are dependent on geographic locations. In industrialized areas, incidence rates range from 0.2 to 3 cases per million, given their increased exposure to asbestos which is the known main cause of peritoneal mesothelioma. Other linked exposure risks include, exposure to erionite, thorotrast, mica and radiation [1, 2]. Our patient had a history of working in a factory that manufactured wire hangers. This could have been a potential source of asbestos exposure. Patient presentation is often vague, symptoms include abdominal pain, ascites, shortness of breath, weight loss, anorexia, diarrhea and vomiting. Abdominal CT scan is often relied on to assist with localization of a mass for fluid sampling as Peritoneal malignant mesothelioma is typically diagnosed through fluid cytology [3]. The World Health Organization (WHO) classifies malignant peritoneal mesothelioma into three histologic subtypes: epithelioid, sarcomatoid and biphasic/mixed [2]. Pathologic evaluation of our patients’ specimen displayed tubulopapillary architecture with monomorphic epithelioid cytomorphology. Tumor noted to penetrate through the ileocecal muscularis propria and submucosa. In addition, immunohistochemical analysis showed strong positive reactions with Calretinin, CK5, CK6, WT-1 and D2–40. These findings supported a diagnosis of diffuse peritoneal malignant mesothelioma, epithelioid subtype which is the most common [2]. Treatment of malignant peritoneal mesothelioma involves neoadjuvant cytoreductive surgery in combination with hyperthermic intraperitoneal chemotherapy with cisplatin and doxorubicin. These perioperative treatments are followed by adjuvant intraperitoneal paclitaxel and second-look cytoreduction [4, 5]. Prognosis of peritoneal mesothelioma is dictated by many factors including completeness of cytoreduction and patient gender. Prognosis is improved in female gender, those who receive intraperitoneal chemotherapy, and in patients who receive cytoreduction. Using a multimodal treatment approach has been reported to result in a median survival of 4–5 years [6]. Given our patient’s atypical presentation with severe small bowel obstruction he was taken for emergent surgical resection. This case points to the fact that malignant peritoneal mesothelioma may be a differential diagnosis in a patient with a small bowel obstruction. Timely and accurate diagnosis and staging can allow for proper treatment protocols and increased longevity after curative surgery. Our patient is currently receiving immunotherapy with nivolumab and ipilimumab due to refusal of chemotherapy treatment. Currently, there has been no evidence of distant metastatic disease after repeat CT and positron emission tomography scans; however, he was found to have mass in the right lateral anterior abdominal wall and peritoneum.

## CONFLICT OF INTEREST STATEMENT

None declared.

## FUNDING

None.
